# Multicenter double blind trial of autologous bone marrow mononuclear cell transplantation through intracoronary injection post acute myocardium infarction – MiHeart/AMI study

**DOI:** 10.1186/1745-6215-9-41

**Published:** 2008-07-03

**Authors:** Hans FR Dohmann, Suzana A Silva, André LS Sousa, Alcione MS Braga, Rodrigo VC Branco, Andréa F Haddad, Mônica A Oliveira, Rodrigo C Moreira, Fabio AA Tuche, Cíntia M Peixoto, Bernardo R Tura, Radovan Borojevic, Jorge P Ribeiro, José C Nicolau, Antonio C Nóbrega, Antonio CC Carvalho

**Affiliations:** 1 Departamento de Pesquisa Clínica, Centro de Ensino e Pesquisa do Pró-Cardíaco/PROCEP, Rio de Janeiro, Brasil; 2 Departamento de Administração e Planejamento em Saúde, Escola Nacional de Saúde Pública Sérgio Arouca/FioCruz, Rio de Janeiro, Brasil; 3 Departamento de Clínica Médica, Universidade Federal do Rio de Janeiro, Rio de Janeiro, Brasil; 4 Departamento de Pesquisa Clínica, Instituto Nacional de Cardiologia/INC, Rio de Janeiro, Brasil; 5 Departamento de Embriologia e Histologia, Universidade Federal do Rio de Janeiro, Brasil; 6 Departamento de Cardiologia, Hospital de Clínicas de Porto Alegre, Rio Grande do Sul, Brasil; 7 Departamento de Cardiologia, Instituto do Coração (InCor) do Hospital das Clínicas da Faculdade de Medicina da Universidade de São Paulo, São Paulo, Brasil

## Abstract

**Background:**

Myocardial infarction remains as a major cause of mortality worldwide and a high rate of survivors develop heart failure as a sequel, resulting in a high morbidity and elevated expenditures for health system resources. We have designed a multicenter trial to test for the efficacy of autologous bone marrow (ABM) mononuclear cell (MC) transplantation in this subgroup of patients. The main hypothesis to be tested is that treated patients will have a significantly higher ejection fraction (EF) improvement after 6 months than controls.

**Methods:**

A sample of 300 patients admitted with ST elevation acute myocardial infarction (STEMI) and left ventricle (LV) systolic dysfunction, and submitted to successful mechanical or chemical recanalization of the infarct-related coronary artery will be selected for inclusion and randomized to either treated or control group in a double blind manner. The former group will receive 100 × 10^6 ^MC suspended in saline with 5% autologous serum in the culprit vessel, while the latter will receive placebo (saline with 5% autologous serum).

**Implications:**

Many phase I/II clinical trials using cell therapy for STEMI have been reported, demonstrating that cell transplantation is safe and may lead to better preserved LV function. Patients with high risk to develop systolic dysfunction have the potential to benefit more. Larger randomized, double blind and controlled trials to test for the efficacy of cell therapies in patients with high risk for developing heart failure are required.

**Trial Register:**

This trial is registered at the NIH registry under the number NCT00350766.

## Background

Acute myocardial infarction (AMI) is a major cause of mortality worldwide. Many survivors of AMI develop heart failure, associated with a high morbidity that worsens their quality of life and generates elevated expenditures for health system resources.[1].

Heart Failure (HF), which has ischemic cardiomyopathy as its most frequent cause, is an epidemic disease of this century. It affects from 2 to 4 million people in the United States (US), and 15 million people around the world.[2] HF in Brazil generates more than 260.000 hospitalizations per year, representing the fourth major cause of hospitalization (2.60% of all hospitalizations in 2007) with an in hospital mortality of 8.1%.[3].

Different medical therapies are efficient for patients who develop systolic dysfunction after AMI, as ACE inhibitors and beta blockers, which aim to improve the infarct repairing response. Recently, cell therapy has appeared as a new perspective. Growing evidence suggests benefits of cell therapies in repair or regeneration the myocardium, and several types of cells have been used. However, cellular and molecular mechanisms responsible for the ventricular function improvements, which have been reported in most of the studies, are still poorly understood. Among other questions, the best pathway to introduce stem cells into the heart has been investigated for the treatment of ischemic cardiomyopathy.

To our knowledge, five other clinical trials of autologous bone marrow (ABM) mononuclear cell (MC) transplantation through intracoronary injection after AMI were published, with more than 300 patients tested and followed for at least 1 year. [4-10] Importantly, no adverse effects associated to the injection procedure were reported.

Even though these are still preliminary data, the results have led us to investigate cell therapy as an alternative treatment of ischemic cardiomyopathy following acute myocardial infarction.

### Hypothesis

The main hypothesis of this trial is that patients submitted to autologous bone marrow stem cell transplantation will present, on average, at least 5% higher EF in comparison with the placebo group after 6 months follow up.

## Methods

This research protocol integrates the Multicenter Randomized Trial of Cell Therapy in Cardiopathies (MiHeart Study) which has been already published.[11] MiHeart Study is divided in four main branches: Dilated Cardiomyopathy; Chagas Disease; Chronic Ischemic Cardiomyopathy and Acute Myocardial Infarction. Here we detail the protocol of the Acute Ischemic Cardiomyopathy branch which was approved by the Institutional Review Board/Ethics Committee at each center including the Anchor Center (PROCEP, Rio de Janeiro) and by the Brazilian National Ethics Council for Human Research (CONEP, Brasilia) in accordance with the principles outlined in the Declaration of Helsinki and National Health Council Resolution n° 196/96.

### Study design

Multicenter, double blind, randomized and placebo controlled trial.

### Population and enrollment

Three hundred patients with ST elevation AMI (STEMI) submitted to successful mechanical (TIMI flow grade 3) or chemical recanalization of the culprit coronary artery up to 24 hours after symptoms onset, and presenting EF ≤ 50% will be selected for inclusion.

Patients will be eligible if presenting all the following characteristics: [1] STEMI; [2] age between 30 and 80 years; [3] EF ≤ 50% (Simpson) and [4] regional dysfunction in the infarct-related area, measured before cells injection. Among patients submitted to fibrinolytic therapy, the angioplasty of the related artery should be done up to 72 hours after fibrinolysis. All patients will be treated with Liberte™ stents, generously donated by Boston Scientific Corp., Natick, Massachusetts.

Patients will be excluded if presenting any of the following: obstruction >50% on Left Main Coronary Artery or multivessel coronary disease, indicating the need for CABG (Coronary Arterial Bypass Grafting); coronary anatomy presenting no need for angioplasty with stent implant; final diastolic pressure of left ventricle (LV) higher than 30 mm Hg during initial ventriculography; cardiac arrest or Killip IV at admission with the need of ventilatory support; cardiogenic shock persisting up to the third day after symptoms onset; mechanical complications of AMI; significant valve disease; chronic use of immunosuppressant agents; >2,0 mg/dl creatinine or previous dialysis treatment; sepsis; sustained ventricular tachycardia after 48 hours post-AMI; illicit drugs abuse or alcohol abuse (based on DSM IV); myocarditis; active liver disease; severe COPD (Chronic Obstrutive Pulmonary Disease); hematological disease, neoplasm, bone disease or haemostatic disturbances; inflammatory disease or chronicle infectious disease; presence of definitive implantation of a cardiac pacemaker or cardiac defibrillator or any co-morbidity, with survival impact in 2 years.

Patients will be included only after signing Informed Consent form, written in accessible language and with information about all relevant aspects regarding the research protocol.

### Randomization

A computer-random number generator will perform randomization procedure in the day of injection as previously published in Trials.[11] Briefly, software was created in R version 1.9.0 to specifically generate the randomization sequence for the study. It is known that a simple randomization is sufficient and efficient to render the study groups homogeneous for known and unknown factors in large scale clinical trials. To insure homogeneity in the two arms of the study we adopted block randomization. We will use randomization by variable size block (blocks of 2, 4 or 6 patients). Patients will be randomized after bone marrow aspiration and only the hematologist responsible for cell separation will have the login and password to randomize the patient online. According to the assigned group, darkened syringes containing the mononuclear cell fraction or saline with 5% autologous serum will be then prepared and sent for injection.

### Cell transplantation Phase

#### ✓ Bone marrow aspiration procedure

About 100 ml of bone marrow aspirate will be harvested from iliac crest between the 5^th ^and 7^th ^day after myocardial reperfusion therapy under local anesthesia and sedation at an intensive care unit or surgical center. The aspiration procedure will be done by using a 1.8 mm Jamshidi needle.

ABMMC will be isolated by density gradient centrifugation on Ficoll- Paque™ plus^® ^(Amersham Biosciences) and manipulated under aseptic conditions. 100 × 10^6 ^cells will be resuspended in 10 ml saline solution with 5% autologous serum and filtered through 100 μm nylon mesh to remove cell aggregates for injection. A fraction of the cell suspension will be used for cell counting and viability testing with trypan blue exclusion. Cell viability must be above 90% to be injected. Post-hoc characterization of leukocyte differentiation markers by flow cytometry (table 1) and functional assays will be done on another fraction of cells. This last evaluation concerns clonogenic capacity of marrow progenitors by colony-forming assays (granulocyte-macrophage colony-forming units – CFU-GM, vascular-endothelial cell colony-forming unit – CFU-VE, and fibroblast colony-forming assay – CFU-F), as previously described.[12,13]

**Table 1:** Post-hoc characterization of leukocyte differentiation markers

**Phenotype**	**Intended designation**
**1**. CD45^lo^CD34^+^	ISHAGE protocol
**2**. Lin^-^CD45^lo^CD34^+^	Hematopoietic progenitors
**3**. Lin^-^CD45^lo^CD34^+^CD133^+^CD117^+^	Hematopoietic stem cells*
**4**. Lin^-^CD45^-^CD133^+^CD31^+^CD144^+^KDR^+^	Endothelial precursor*
**5**. Lin^-^CD45^-^CD133^-^CD31^+^CD144^+^KDR^+^	Endothelial cell*
**6**. CD45^+^CD10^+^CD33^+^HLA-DR^-^CD45RA^-^	Myeloid precursor*
**7**. CD45^+^CD10^+^CD33^-^HLA-DR^+^CD45RA^+^	Lymphoid precursor*
**8**. CD45^-^CD133^+^CD146^+^CD144^+^CD166^+^	Mesenchymal progenitor*
**9**. CD45^-^CD133^-^CD146^+^CD73^+^CD166^+^	Mesenchymal cell*
**10**. CD45^+^CD10^+^CD34^+^CD19^-^CD38^-^TdT^+^IgM^-^	pre-pro-B intermediary
10.1. CD45^+^CD10^+^CD34^+^CD19^+^CD38^-^TdT^+^IgM^-^	pro-B/pre-B I intermediary
10.2. CD45^+^CD10^+^CD34^-^CD19^+^CD38^-^TdT^-^IgM^-^	Pre-B II intermediary
10.3. CD45^+^CD10^-^CD34^-^CD19^+^CD38^+^TdT^-^IgM^+^CD24^hi^	Immature B LΦ
10.4. CD45^+^CD10^-^CD34^-^CD19^+^CD38^+^TdT^-^IgM^+^	Naïve B LΦ
10.5. CD45^+^CD10^-^CD34^-^CD19^+^CD38^+^TdT^-^IgM^+^CD24^lo^CD69^+^	Mature B LΦ
**11**. CD45^+^CD34^+^CD38^-^CD1a^-^CD3^-^CD4^-^CD8^-^	LΦ-T DN1
11.1. CD45^+^CD34^+^CD38^+^CD1a^-^CD3^-^CD4^-^CD8^-^	LΦ-T DN2
11.2. CD45^+^CD34^+^CD38^+^CD1a^+^CD3^-^CD4^-^CD8^-^	LΦ-T DN3
11.3. CD45^+^CD34^+^CD38^+^CD1a^+^CD3^lo^CD4^+^CD8^-^	LΦ-T DN4-ISP
11.4. CD45^+^CD34^+^CD38^+^CD3^-^CD4^+^CD8^+^	LΦ-T DP
11.5. CD45^+^CD34^+^CD38^+^CD3^+^CD4^+^CD8^+^	LΦ-T DP-CD3
**12**. CD45^+^CD34^+^CD3^+^CD4^+^CD8^-^	LΦ-T CD4
**13**. CD45^+^CD34^+^CD3^+^CD4^-^CD8^+^	LΦ-T CD8
**14**. CD45^+^CD122^+^CD56^+^CD3^-^	NK cells
**15**. CD45^+^CD66acde^+^	Neutrophils/Basophils
**16**. CD45^+^CD41a^+^	Megakaryocytes
**17**. CD45^+^CD66acde^-^CD41a^-^CD14^+^CD16^+^	Monocytes/Macrophages

*The intended designation to some phenotypes will be more deeply explored with additional markers like CD117, CD31, CD105, CD106, CD166 and others of interest. Besides, cell subpopulations also will be sorted to perform supplementary function assays.

An out of hospital independent team at each site, composed by 2 biologists trained by the anchor center, will be the responsible for cell separation and preparation of the solution according to the randomization result. Control group will receive a placebo saline solution containing autologous blood serum and treated group will receive a saline solution containing ABMMC. The solutions containing cells or placebo will be placed in opaque syringes (covered in 50% insulfilm), reinforcing the blind application, for injection.

In order to guarantee the quality of the material, imunophenotyping and clonogenic capacity analysis will be all done at a Core Lab as detailed in the appendix [see Additional file 1]. Cells will be kept in appropriate conditions and sent to the cells core lab for these analyses.

Exceeding material and cells collected from patients in the placebo group will be stored, duly frozen, until the end of the trial.

Bone marrow collected from patients included in control group will be used for phenotyping and cell analysis, which will be correlated with patients' clinical evolution.

### Cell transplantation

Cell delivery is predicted to be performed about 6 hours after bone marrow cell harvesting. Arterial accesses will be performed using femoral or radial approach. All patients will receive 10,000 IU of heparin IV after sheet insertion. When non-infarct related vessel intervention is planned, it will be performed before cell transfer. Electrocardiography, pulse oximetry, and vitalsigns will be monitored throughout the procedure, as well as symptoms.

A 6-F guiding catheter will be placed at the ostium of the infarct-related coronary artery. After positioning the catheter inside the coronary ostium, a dosage of isossorbide mononitrate will be administered (total of 1 ml = 10 mg). Then, a coronary will be performed in a calibrated system in order to visualize the coronary blood flow before injection. At least one projection for evaluating "TIMI frame count" will be performed with a 30 frame/sec filming, attempting to visualize the distal coronary bed.

After TIMI 3 flow scrutiny, an over-the-wire (OTW) balloon catheter (Maverick^® ^Over-The-Wire balloon, Boston Scientific, Natick, MA, USA) will be positioned inside the previously implanted stent in the culprit vessel to transiently interrupt anterograde blood flow during infusions through a stop-flow technique. The use of the *over-the-wire *balloon with over 0.5 mm of the reference lumen diameter in a low pressure (< 4ATM) is recommended. The ideal balloon extent is 9 mm, limited to the edge of the previously implanted stent.

Cells solution will be infused through the central lumen of the balloon catheter, in about 40 seconds, during 3 coronary occlusions, each lasting 2 – 3 minutes, followed by 2 minutes of balloon deflation.

A final coronary angiography will be performed at the end of all procedures as control in order to ascertain TIMI frame count pre and post PCI and cell transfer.

#### ✓ Baseline exams (before bone marrow aspiration) and follow up

The chronogram of the study is shown in table 2. Briefly, patients will be submitted to a clinical evaluation at hospital admission and clinical data regarding blood pressure, heart rate and Killip and Kimball classification will be stored. Patients will be standardly treated with chemical or mechanical reperfusion according to the local center availability. A coronary angiography and an invasive ventriculography will be performed in all patients before angioplasty in order to evaluate inclusion/exclusion criteria. Patients submitted to fibrinolysis must have the angioplasty of the infarct-related coronary artery performed up to 48 hours after reperfusion. Laboratorial dosage will be also performed as shown in table 2.

**Table 2:** Chronogram of the study

Informed Consent	□											
Randomization				□								

**Exams/Visits**					**After Injection**							
										
	Baseline	pos-reperfusion	3^rd^–5^th ^day of reperfusion	5^th ^to 7^th ^day	Up to 1 h	12 h	24 h	6^th ^to 9^th ^day	1 month	3 months	6 months	1 year

Medical visit	□		□						□	□	□	□
ALT, AST, AP, PT, aPTT and INR			□									
Total Cholesterol, LDL-cholesterol, HDL-cholesterol and Triglycerides	□											
Full blood panel, Glucose, Urea, Creatinine, Na+, K+, CRP			□				□		□		□	
CK-MB mass, Troponin I	□		□	□		□	□					
Electrocardiogram	□	□	□		□	□	□		□	□	□	□
Echocardiogram			□						□	□	□	□
Cardiac MRI								□			□	
Coronary angiography	□										□	
Cells transplant				□								
Cells sample storage				□								
SF-36, Minnesota and Seattle QOL			□						□		□	□
QALY Evaluation									□		□	□

ALT and AST: alanine and aspartate aminotransferases, respectively; AP: alkaline phosphatase; INR: international normatized ratio; PT: prothrombin time; aPTT: activated partial thromboplastin time; CRP: C-reactive protein; MRI: magnetic resonance image; QOL: quality of life questionnaires; QALY: quality adjusted life year.

Three to five days after reperfusion therapy (mechanical or chemical), patients will be submitted to another clinical evaluation, including an EKG, an echocardiogram and laboratory tests, from which results will be used as baseline data for the research protocol, before injection procedure. A new CK-MB mass and troponin dosage will be performed in the morning preceding the injection procedure and timely in the 24 hours following the injection.

The baseline cardiac magnetic resonance imaging (MRI) will be performed between day 6 and 9 after the AMI-related coronary artery recanalization. This exam may be performed either in the hospital or at a reference hospital. All imaging exams will be evaluated off line by the core lab specified in the appendix [see Addition file 1].

Hospital discharge is predicted 24 hours after cell transplantation unless otherwise indicated, making a total length of stay of approximately 7 days. Patients will be provided with clopidogrel, aspirin and pravastatin during the entire study and treated at the discretion of the attending physician.

Patients will be followed for 1 year after the procedure according to the chronogram of the study shown in table 2. All imaging exams will be stored and sent to the core lab for independent analysis as specified in the appendix [see Addition file 1].

Cardiac MRI will be done by the local centers with commercially available software, electrocardiographic triggering, and cardiac-phased array coil. LV function will be assessed by the use of steady-state free-precession gradient-echo sequence (SSFP). Eight to twelve short axis slices will be acquired during a 10–15 seconds breath-holding allowing for coverage of the entire LV. Additionally, 3 long-axis views will also be acquired: 2-chamber and 4-chamber views and left ventricle out flow tract. Regional LV function will be assessed by measuring systolic wall thickening in the infarct region, the adjacent border zone and remote myocardium and semi-quantitatively assessed, using a 17-segment model[14].

Transmural extent of late hyper-enhancement within each segment will be graded according to the following classification: 0–25%, 26–50%, 51–75%, and more than 75% hyper-enhancement. Segmental functional recovery is defined as an increase from hypokinetic to normokinetic, from akinetic to hypokinetic or normokinetic, or from dyskinetic to akinetic, hypokinetic, or normokinetic. Infarct size will be measured by planimetry.

Microvascular obstruction will be defined on late enhanced images taken early (within 2–5 min) after injection of 0.20 mmol/kg of gadopentetate dimeglumine (Gd-DTPA), in the LV short and long axis at the same locations used for cine-MRI–as a dark, subendocardial zone within the infarct area.

For assessment of global and regional LV function and calculation of LV mass, endocardial and epicardial borders will be traced in end-diastolic and end-systolic short-axis slices. LV end-diastolic and end-systolic volumes (LVEDV and LVESV) will be calculated and indexed to body-surface area. All MRI studies will be independently analyzed on an off-line workstation, core laboratory as defined in the appendix [see Additional file 1].

### Sample size

Sample size calculation was performed according to the following assumptions:

▪ Based on Pró-Cardíaco Hospital and InCor's coronary care databank, after selecting AMI patients Killip I; II or III, we have concluded that the left ventricle EF presented a Gaussian distribution with an average of 37.35% and standard deviation of 7.9%).

▪ An expert panel has evaluated that an improvement of +5% (relative gain of +15.9%) on the EF would represent a significant clinical improvement for the patient.

Considering as clinically beneficial a 5% increase in EF% with a standard deviation of 5%, 143 patients per arm are needed to detect a significant difference between groups with 95% confidence and 80% power. Considering a 5% loss during follow up, we estimated 150 patients per arm.

### End points and statiscal analysis

The primary endpoint of this trial is the global left ventricular EF change from baseline to 6 month follow-up.

The following secondary endpoints will be evaluated for up to 1 year of follow up: major cardiovascular events (all-cause mortality, acute myocardial infarction, stroke and hospital admission due to cardiovascular cause); reintervention of the AMI-related artery and of non-related arteries; by MRI regional wall motion, wall thickening, and volume of late contrast enhancement; quality of life assessment using the Short-Form 36, Minnesota Living with Heart Failure and Seattle Angina questionnaires; cost-effectiveness and cost-utility evaluation of ABMMC implant versus conventional treatment.

All outcomes will be analyzed by intention to treat.

Primary outcome will be analyzed by comparing EF, measured by MRI, between groups, using student t test or Mann-Whitney according to the sample distribution. Two evaluations are programmed:

1. Interim analysis, which will use data related to patients 1 to 150;

2. Final analysis, which will use data related to all 300 patients.

Secondary outcomes will only be evaluated after the main final analyses. Mortality and functional class improvement will be analyzed by non-corrected chi-square test. Continuous variables with normal distribution will be analyzed by t student test and ANOVA for repeated measures. Continuous variables with asymmetric distribution and quality of life scores will be analyzed by Mann-Whitney test and ANOVA for repeated measures.

The frequency of serious adverse events (which brings eminent risk of death or permanent disability) will be analyzed by chi-square or Fisher's test.

The trial can be interrupted after interim analysis; using the O'Brien and Fleming rule for two sequential tests and the 5%, in which the null hypothesis is rejected if:

• p ≤ 0,0050 in the first analysis;

• p ≤ 0.04806 in the final analysis.

### Study organization and monitoring

The MiHeart trial is supervised by an executive committee and coordinated by a study coordination committee. Members of both committees are listed in the appendix [see Additional file 1]. The steering committee is responsible for design and conduct of the study. An independent data and safety monitoring committee monitors the patient safety as the study progresses. Data will be stored at both a CRF and at a central database through e-CRF fulfillment as previously published, and used as tools for data monitoring by comparing with the original reports.[11].

### Current status

The names of all participating centers and their representatives are provided in the appendix [see Additional file 1]. Recruitment commenced in August 2006 and is expected to be completed in August 2009. Analysis and reporting is to be completed by August 2010.

## Discussion

Stem cell therapy still remains as a promising modality to occupy a real gap of conventional AMI therapy, especially when this acute event is followed by LV remodeling and chronic heart failure. There is increasing evidence that stem cells contribute to the regeneration of cardiac tissue as a natural healing process following STEMI, thus opening new possibilities for the treatment of this condition.

The safety of ABMMC intracoronary transplantation after AMI has been shown previously and although data supporting the improvement of ventricular function are conflictant, [15-17] the preliminary results regarding regional contractility and infarct size of most of these studies are promising.[5,8,15,17,18] Additionally, it should be noted that many patients included in these trials had preserved ventricular function, thus encompassing the subgroup most unlikely to benefit from cell therapy.

As demonstrated in the REPAIR AMI trial, [5] patients with the worst EFs show greater benefit from cell transplantation. Even though these analyses were underpowered to draw meaningful conclusions about degree of EF impairment at baseline and follow up and timing of injections, these initial clinical trials have shown major cardiovascular adverse events reduction at 1 year in patients submitted to intracoronary infusion of ABMMC,[19] encouraging further phase III trials.

Nevertheless, the current evidence is insufficient to prove that BM cell therapy after an acute MI can provide additional beneficial effects on LV function beyond those achieved by early reperfusion therapy.

Therefore, we have designed a randomized large placebo-controlled trial, to inject ABMMC using the intracoronary route in patients with left ventricular dysfunction following AMI who were successfully treated with either chemical or mechanical reperfusion. The purpose of this study is to test for additional benefits of stem-cell therapy in this scenario where patients have ventricular dysfunction due to irreversible myocardial damage. If this assumption proves to be correct stem cell therapy will be a valid strategy for treating this obscure and harmful condition, reducing morbidity, mortality, heart transplantation indication, costs of the treatment and improving life quality.

## Competing interests

The authors declare that they have no competing interests.

## Authors' contributions

HD is the PI of this study and AC is the study coordinator. All authors contributed to the development of the protocol and were involved in the writing of the manuscript.

## Supplementary Material

Additional file 1 Appendix. The data provided describes all committees involved in the coordination of this study, as well as core lab and collaborating centers members. Click here for file
