# Supplementary material for: Multicenter double blind trial of autologous bone marrow mononuclear cell transplantation through intracoronary injection post acute myocardium infarction – MiHeart/AMI study
Source: Trials. 2008 Jul 3;9:41. doi: 10.1186/1745-6215-9-41 (PMC2486263; doi:10.1186/1745-6215-9-41)
Supplement: Additional file 1 — Appendix. The data provided describes all committees involved in the coordination of this study, as well as core lab and collaborating centers members. [file 1745-6215-9-41-S1.doc]

**Appendix A**

This trial is divided in three centers: (1) Coordinating Center – Organ composed by the Ministry of Health which will be responsible for the general organization, including randomization, monitory, creation and maintenance of database and technical assistance. (2) Anchor Center – a tertiary Hospital focused on cardiology and research, including professionals recognized for stem cell research leadership and research infrastructure in this area, in charge of the design of the protocol and its execution. (3) Collaborating Centers: hospitals that have an established cardiology center, which is capable of properly enrolling patients and providing their follow up during the study period.

Centers were qualified according to Ministry of Health (MS) and Ministry of Science and Technology’s (MCT) specifications on July 15^th^, 2004, number 10.04.284.00.

(<http://www.finep.gov.br/fundos_setoriais/outras_chamadas/resultados/resultado_celulas_tronco.PDF>).

Four Institutions were qualified for heading the Multicenter Randomized Trial of Cell Therapy in Cardiopathies as Anchor Centers – (Dilated Cardiomyopathy - Instituto Nacional de Cardiologia/INC; Chagas Disease - Hospital Santa Izabel; Chronic Ischemic Cardiomyopathy - Instituto do Coração/USP-InCor; Acute Myocardial Infarction - Instituto de Biofísica/UFRJ-IBCCF and Centro de Ensino e Pesquisa do Pró-Cardíaco/PROCEP).

**Appendix B**

**Executive committee –** Composed by Ministry of Health (MS); Ministry of Science and Technology (MST) and The Financing Agency for Studies and Projects (FINEP) representatives: Reynaldo F. N. Guimarães - MS; Suzane J. Serruya – MS; Angélica Pontes – MS; Ana Paula Correa – MS; Maura F. Pacheco – FINEP; Sandra Carvalho – FINEP.

Steering committee - Antonio C. C. Carvalho, MD, PhD (chairman) – INC, Rio de Janeiro; Hans F. R. Dohmann, MD, MSc (principal investigator for MiHeart/AMI) – PROCEP, Rio de Janeiro; Regina C. S. Goldenberg, PhD – UFRJ/IBCCF, Rio de Janeiro; Ricardo R. Santos, MD, PhD – FioCruz, Bahia; Gilson S. Feitosa, MD, PhD – Hospital Santa Izabel, Bahia; Augusto Bozza, MD, PhD - INC, Rio de Janeiro; Helena Martino, MD - INC, Rio de Janeiro; José E. Krieger, MD, PhD – InCor, São Paulo; Noedir Stolf, MD, PhD - InCor, São Paulo; José C. Nicolau, MD, PhD – InCor, São Paulo

**Appendix C**

Coordinating Center: Antonio C. C. Carvalho, MD, PhD (chairman) – INC, Rio de Janeiro

**Leading Center:** Hans Fernando R. Dohmann, MD, MSc (principal investigator) –PROCEP, Rio de Janeiro; Regina C. S. Goldenberg, PhD – UFRJ/IBCCF, Rio de Janeiro

Collaborating Centers:

1. João Alexandre Assad, MD – Hospital Pró-Cardíaco, Rio de Janeiro;
2. Constantino R Constantine, MD, PhD – Hospital Cardiológico Costantini, Curitiba;
3. Gilson S Feitosa, MD, PhD – Hospital Santa Izabel, Bahia;
4. Adrian P M Kormann, MD – Hospital Santa Isabel, Blumenau;
5. João Moraes Jr., MD – Hospital Agamenon Magalhães, Recife;
6. Augusto C. Cassano, MD – Hospital Municipal do Andaraí, Rio de Janeiro;
7. Jorge P. Ribeiro, MD, PhD – Hospital de Clínicas, Porto Alegre;
8. Marco A. Mattos, MD, PhD – Instituto Nacional de Cardiologia, Rio de Janeiro;
9. Rogério S. Leite, MD, PhD – Instituto de Cardiologia, Rio Grande do Sul;
10. Vinício E. Soares, MD, MSc – Hospital Municipal Miguel Couto, Rio de Janeiro;
11. Anis Rassi, MD, PhD – Hospital Anis Rassi, Goiania;
12. Antonio C. C. Carvalho, MD, PhD – Hospital São Paulo, São Paulo;
13. Helio J Castello, MD, MSc – Hospital Bandeirantes, São Paulo;
14. Adriano M Caixeta, MD, PhD – InCor, Distrito Federal;
15. José A. Marin-Neto, MD, PhD – Hospital das Clínicas, Ribeirão Preto;
16. Leonardo Vieira, MD – InCor, São Paulo;
17. Itamar R. Oliveira, MD – InCor, Natal;
18. Alfredo B. Teixeira, MD – Hospital Geral de Bonsucesso, Rio de Janeiro;
19. Marco A. C. Araújo, MD – Hospital Municipal Souza Aguiar, Rio de Janeiro.

All centers are in Brazil.

**Appendix D**

Core laboratory for in vitro studies on cell material: Radovan Borojevic, PhD, Rosana B. Carias, PharmD, MSc and Hamilton S. Junior, PhD – Excellion Serviços Biomédicos S/A, Petrópolis.

**Appendix E**

Core laboratory for MRI: Carlos E. Rochitte, MD, PhD – InCor, São Paulo and Amarino O. Junior, MD – Hospital Pró-Cardíaco, Rio de Janeiro

**Appendix F**

Randomization and data management: Bernardo Tura, MD, PhD and Eduardo Hill, PhD – Trial Coordination Center, Rio de Janeiro.
